# Evolutionary dynamics and transcriptional diversification of GAD gene family in plants: a pan-species perspective in *silico* analysis

**DOI:** 10.1186/s12870-025-07691-4

**Published:** 2025-11-22

**Authors:** Yunxia Niu, ZiHao He, Xuanwen Yang, Shance Niu

**Affiliations:** 1 School of Vocational Education, Tianjin University of Technology and Education, Tianjin, 300222 China; 2 College of Horticulture, State Key Laboratory of North China Crop Improvement and Regulation, Hebei Agricultural University, Baoding, 071000 China; 3 State Key Laboratory of Tropical Crop Breeding, Tropical Crops Genetic Resources Institute, Chinese Academy of Tropical Agricultural Sciences, Haikou, 571101 China

**Keywords:** Glutamate decarboxylase, Plant evolution, Tandem duplication, Expression pattern, Cis-elements, Intron-mediated enhancement, GABA signaling

## Abstract

**Background:**

Glutamate decarboxylase (GAD) catalyzes the biosynthesis of γ-aminobutyric acid (GABA), a key metabolite and signaling molecule in plant growth and stress responses. However, the evolutionary history and regulatory complexity of GAD genes across plant lineages remain poorly understood.

**Results:**

We identified 182 GAD genes from 31 plant species, including basal Embryophyta, monocots, and eudicots. Phylogenetic analyses grouped these genes into three major subfamilies (A, B, C), with subfamily A confined to early land plants, suggesting an ancient evolutionary origin. Tandem duplication was the dominant mechanism for expansion in eudicots, whereas annotated splice isoforms suggest potential contributions to transcriptomic diversity in half of the species analyzed. Structural characterization revealed high motif conservation but also specific domain innovations, such as heavy-metal-associated domains in flax. Promoter and intron analyses showed a high abundance of light-, ABA-, MeJA-, and anaerobic-responsive elements. Notably, intron length correlated positively with gene expression across tissues, suggesting a key role for intron-mediated enhancement. RNA-seq data from *Dendrobium catenatum*, *Tagetes erecta*, *Arabidopsis*, rice, and cotton revealed dynamic tissue-specific expression patterns, with certain ancestral GAD genes displaying low, restricted expression, and newer paralogs showing broader profiles.

**Conclusions:**

The GAD gene family exhibits lineage- and tissue-specific expression patterns rather than strict subfamily-level functional divergence. Intronic length and regulatory motif content strongly correlate with transcriptional activity. This work offers novel insights into the evolution, expression regulation, and functional diversification of GAD genes across plants, with practical implications for crop improvement and stress resistance.

**Supplementary Information:**

The online version contains supplementary material available at 10.1186/s12870-025-07691-4.

## Introduction

γ-Aminobutyric acid (GABA), a non-proteinogenic amino acid, is widely distributed among microorganisms, plants, and animals, where it fulfills a range of essential physiological functions [1–4]. Glutamate decarboxylase (glutamic acid decarboxylase; GAD; EC 4.1.1.15) is an enzyme that irreversibly catalyzes the conversion of L-glutamate to GABA in the presence of the cofactor pyridoxal 5’-phosphate (PLP) [5]. Plant GADs exhibit distinct regulatory mechanisms not observed in microbial or animal systems, notably through a Ca²⁺-dependent interaction with calmodulin (CaM) [6, 7]. Two major regulatory pathways have been described: (1) a pH-dependent pathway, where maximal activity occurs at acidic pH (~ 6.0); and (2) a Ca²⁺/CaM-dependent pathway, which enhances GAD activity near neutral pH (~ 7.5) [8].

The GABA shunt, which includes GAD, bypasses part of the tricarboxylic acid (TCA) cycle and links primary metabolism to stress responses. This pathway involves (1) the conversion of GABA to succinic semialdehyde by GABA transaminase, and (2) the oxidation of succinic semialdehyde to succinate by succinic semialdehyde dehydrogenase [9, 10]. This alternative route contributes to carbon/nitrogen balance and is interconnected with both energy metabolism and amino acid biosynthesis [11–15].

In addition to its metabolic roles, GABA functions as a signaling molecule in plants. It modulates the activity of aluminum-activated malate transporters (ALMTs) and regulates diverse developmental and stress-related processes [3, 9, 16]. GAD has been implicated in diverse developmental processes, including stem elongation in rice [7, 17], vascular and hypocotyl development in pine [18], and adventitious root formation in poplar [19]. However, studies in *Solanum lycopersicum* have reported that strong down-regulation of GADs had little impact on overall plant growth, fruit development, or primary fruit metabolism under normal growth conditions [20]. Furthermore, GAD mediates responses to biotic and abiotic stresses, such as pathogen infection (*Pseudomonas syringae*, *Ralstonia solanacearum*) in tomato [21, 22]; cadmium (Cd²⁺) toxicity in maize [23], phosphorus deprivation in *Arabidopsis* [24], and osmotic and hypoxic stress. For instance, NaCl stress downregulates AtGAD1 while upregulating AtGAD2 and AtGAD4 [25]; hypoxia induces AtGAD4 expression specifically [26]; and cold, mechanical injury, and low oxygen increase GAD activity more broadly [27].

In mammals, GABA acts as the principal inhibitory neurotransmitter, contributing to synaptic regulation, brain development, and neural homeostasis [2]. Altered GABA signaling is implicated in a range of disorders, including autism spectrum disorder, schizophrenia, epilepsy, depression and so on [28, 29]; D. Huang et al., [30–33]. These findings suggest that both GAD and GABA are evolutionarily conserved components critical to cellular regulation across kingdoms.

To date, evolutionary analyses of GAD genes in plants have been limited to a few species, such as cotton [30, 34], longan [35], apple [36], and soybean [37]. These studies indicate that, compared to microbes and animals [38, 39], plants often possess expanded GAD gene families—e.g., five members in rice, six in Arabidopsis thaliana, and ten in cotton [10, 34]—reflecting functional divergence and specialization. Comprehensive, cross-species investigations are still lacking but are essential for understanding how GADs have evolved in relation to plant adaptation and development.

Orchidaceae, one of the largest and most morphologically diverse angiosperm families, represents a promising lineage for such evolutionary studies due to its extensive ecological range, specialized floral structures, and high ornamental value [40]. Similarly, marigold (Tagetes erecta L.) is not only an important ornamental plant but also a major source of lutein, a carotenoid pigment used in food, medicine, and feed industries [41–43]. Despite their economic and ecological importance, GAD gene families in these species remain poorly characterized.

In this study, we conducted a genome-wide identification and comparative analysis of GAD genes across 31 representative plant species, with a focus on the expression and evolution of GAD genes in *Dendrobium catenatum* (D. catenatum) and marigold. To gain further insights into conserved and divergent expression patterns, we also analyzed RNA-seq data from three model plants: *Arabidopsis*(*Arabidopsis thaliana*), rice(*Oryza sativa*), and cotton(*Gossypium hirsutum*). The specific objectives of this study were to:

1. Identify and characterize GAD gene families across diverse plant species;
2. Profile gene expression in different tissues of *D. catenatum*, marigold, and model species to reveal functional differentiation;
3. Investigate evolutionary patterns and regulatory divergence among GAD family members.

By integrating gene structure, phylogenetics, and transcriptomic data, this study aims to enhance our understanding of the functional evolution of GAD genes and provide a framework for future investigations into their roles in plant development and stress adaptation.

## Materials and methods

### Identification of GAD family members and data sources

Genomic data for *Arabidopsis thaliana*(TAIR10) (including genome sequences, GFF3 annotation files, gene annotation files, and protein sequences) were obtained from the TAIR database (https://www.arabidopsis.org/). For mostly other plant species, genome assemblies, annotation files (GFF3 annotation files, gene annotation files), and corresponding protein sequences were downloaded from the Phytozome(v13) (https://phytozome-next.jgi.doe.gov/).

The selected species covered a wide phylogenetic range, including algae, early land plants, basal angiosperms, monocots, and eudicots, with genome versions (Phytozome release) specified where applicable:

1. Rhodophyta :*Porphyra umbilicalis* (v1.5);
2. Chlorophyta: *Chlamydomonas reinhardtii*(v6.1), *Dunaliella salina*(v1.0), *Micromonas strain RCC299*(v3.0);
3. Bryophytes: *Marchantia polymorpha*(v3.1), *Physcomitrella patens*(v3.3);
4. Lycophytes: *Selaginella moellendorffii*(v1.0);
5. Basal angiosperms: *Amborella trichopoda*(v2.1)(*Amborellaceae*);
6. Eudicots:
  - Basal eudicots: *Aquilegia coerulea*(v3);
  - Caryophyllales: *Beta vulgaris* (sugar beet)(EL10.2);
  - Rosids(including fabids & malvids): *Vitis vinifera*(v2.1)*(grapevine)*,* Cucumis sativus*(v1)(cucumber), *Phaseolus vulgaris*(v2.0)(common bean), *Linum usitatissimum*(v1.0)(flax), *Salix purpurea*(v5.1), *Theobroma cacao*(v2.0) (cacao), *Lepidium sativum*(v1)(garden Cress), *Brassica rapa* (FPsc variety)(v2.1), *Gossypium hirsutum*(v3.0)(upland cotton), *Citrus sinensis*(v1)(sweet orange);
  - Asterids: *Helianthus annuus*(r1.0)(sunflowers), *Solanum tuberosum*(v6.1)(potato);
7. Monocots: *Oryza sativa*(v3.0)(rice), *Musa acuminata*(v1)(banana), *Brachydium distachyon*(v3.0), *Miscanthus sinensis*(v7.0), *Setaria italica*(v2)(foxtail millet), *Sorghum bicolor*(v5.0)(sorghum).

Additionally, transcriptomic and genomic datasets for several orchid species(Monocots)—including *Dendrobium chrysotoxum* [44], *Dendrobium nobile*(v1) [45], *Dendrobium catenatum*(v1) [46], *Apostasia shenzhenica*(v1) [47], *Dendrobium chrysanthum*(v1)(Niu et al., unpublished)*—*as well as *Tagetes erecta*(v2) (Asterids)(Xin et al., [43]were retrieved from NCBI database(https://www.ncbi.nlm.nih.gov/) and the National Genomics Data Center (NGDC) (https://ngdc.cncb.ac.cn/).

In total, 35 plant species were selected for analysis. To identify candidate GAD gene family members, five well-characterized *Arabidopsis* GAD sequences (*AT2G02010*, *AT5G17330*, *AT2G02000*, *AT1G65960*, *AT3G17760*) and five rice GAD sequences (*LOC_Os03g13300.1*, *LOC_Os03g51080.1*, *LOC_Os04g37460.1*, *LOC_Os04g37500.1*, and *LOC_Os08g36320.3*) were used as query sequences to represent dicotyledonous and monocotyledonous lineages, respectively. Candidate GAD sequences were identified using the BLASTP algorithm implemented in TBtools v2.119 [48], with an E-value threshold of < 1e^-^⁶ and a sequence identity cutoff of >40%. Conserved domain information was obtained from the NCBI CD-Search Tool(https://www.ncbi.nlm.nih.gov/Structure/bwrpsb/bwrpsb.cgi). To further enhance the accuracy and comprehensiveness of domain annotation, InterProScan and Pfam databases were also employed to confirm the presence of conserved functional domains essential for GAD activity.

### Physicochemical Properties, phylogenetic Analysis, and sequence alignments of GAD proteins

All full-length candidate GAD protein sequences, including those from *Arabidopsis* and rice, were aligned using MAFFT with default parameters [49]. The phylogenetic tree was then constructed using the maximum likelihood (ML) method implemented in PhyML 3.0 (http://www.atgc-montpellier.fr/phyml/) [50], with 1000 bootstrap replicates to assess tree reliability. Additionally, branch support was estimated using an approximate likelihood-ratio test (aLRT) inspired by the Shimodaira–Hasegawa-like procedure, with the Whelan and Goldman(WAG) model [50]. The resulting tree was visualized and annotated using FigTree v1.4.4 (http://tree.bio.ed.ac.uk/software/figtree/) and the Interactive Tree of Life (iTOL, https://itol.embl.de/) [51].

The physicochemical characteristics of GAD proteins(Supplementary 1 Table S1)—including instability index (II), isoelectric point(pI), protein length (amino acids, aa), aliphatic index (AI), and grand average of hydropathicity (GRAVY)—were predicted using the ExPASy ProtParam tool (https://web.expasy.org/protparam/).

### Conserved motifs and gene structure analyses

Conserved protein motifs were identified using the MEME Suite (http://meme-suite.org/) [52]. Based on the approach of Huang et al. [30], the maximum number of motifs was set to 15, while all other parameters were kept at their default values. The MEME results were subsequently used for gene structure visualization in TBtools-II [48]. The exons and introns regions of the GAD genes were analyzed and visualized by loading the GFF files to TBtools [48]. TBtools was also employed to generate an integrated schematic representation of phylogenetic relationships, gene structures, conserved domains, and motif compositions among GAD gene family members. Gene duplication types were identified using DupGen_finder-unique (version 1.0.0)(Qiao et al., [53] with default parameters, based on BLASTP (E-value < 1e − 5) and collinearity blocks from MCScanX [54]. Genes were classified into five categories—WGD, tandem, proximal, transposed, and dispersed—following the priority rule WGD > tandem >proximal > transposed >dispersed.

### Chromosomal Distribution, subcellular Localization, and Cis-acting elements analyses

Chromosomal localization of GAD genes across the 31 species was determined using TBtools-II [48], based on genomic annotation information from the corresponding GFF3 files (Supplementary 1 Table S1).

Subcellular localization of GAD proteins was predicted using the WoLF PSORT web server (https://wolfpsort.hgc.jp/)(Supplementary 1 Table S1). To analyze regulatory elements, 2000 bp upstream promoter regions of the GAD genes were extracted, and potential cis-acting elements were predicted using the PlantCARE database [55] https://bioinformatics.psb.ugent.be/webtools/plantcare/html/) (Supplementary 1 Table S2).

Additionally, intronic cis-regulatory elements were identified in representative species including in *Arabidopsis*,* D.catenatum*, cotton, rice, and marigold(Supplementary 1 Table S3). All curated data were visualized using TBtools [48].

### Expression analysis

To investigate the evolutionary expression patterns of GAD genes, transcriptome datasets from *Dendrobium catenatum* and *Tagetes erecta* (marigold) were analyzed. The RNA-seq data for D. catenatum were obtained from Zhang et al. (PRJNA262478) [46], with each sample comprising three biological replicates. For marigold, transcriptome data were retrieved from Xin et al., with five biological replicates per sample (see Sect. 2.1 for dataset details).

To further explore the expression divergence of GAD genes across evolutionary lineages, expression profiles in various tissues of *Arabidopsis*, rice, and cotton were compared (Supplementary 2–4). Gene-level FPKM(fragments per kilobase of transcript per million mapped reads) values were downloaded from the *A. thaliana* RNA-Seq Database (https://plantrnadb.com/). Only datasets with high uniquely mapped read rates and recent sequencing dates were included in the analysis to ensure data quality.

### Statistical analyses

One-way analysis of variance (ANOVA) was performed to assess the statistical significance of expression differences among tissues in *D.catenatum*, marigold, *Arabidopsis* and rice. For *D. catenatum*, post hoc pairwise comparisons were conducted using the Bonferroni correction when homogeneity of variance was satisfied, and Tamhane’s T2 method when variance heterogeneity was detected. For marigold, *Arabidopsis* and rice, Tukey’s multiple comparisons test was applied.

Due to limited sample sizes, anther expression data in *Arabidopsis* and ovule data in rice were excluded from the one-way analysis of variance. All graphs were generated using GraphPad Prism 8 for Windows. Results are presented as mean ± standard error (SE), and statistical significance was defined as *p* <.05.

## Results

### Phylogenetic, subcellular Localization, and physicochemical properties analysis of GADs

**Fig. 1:**
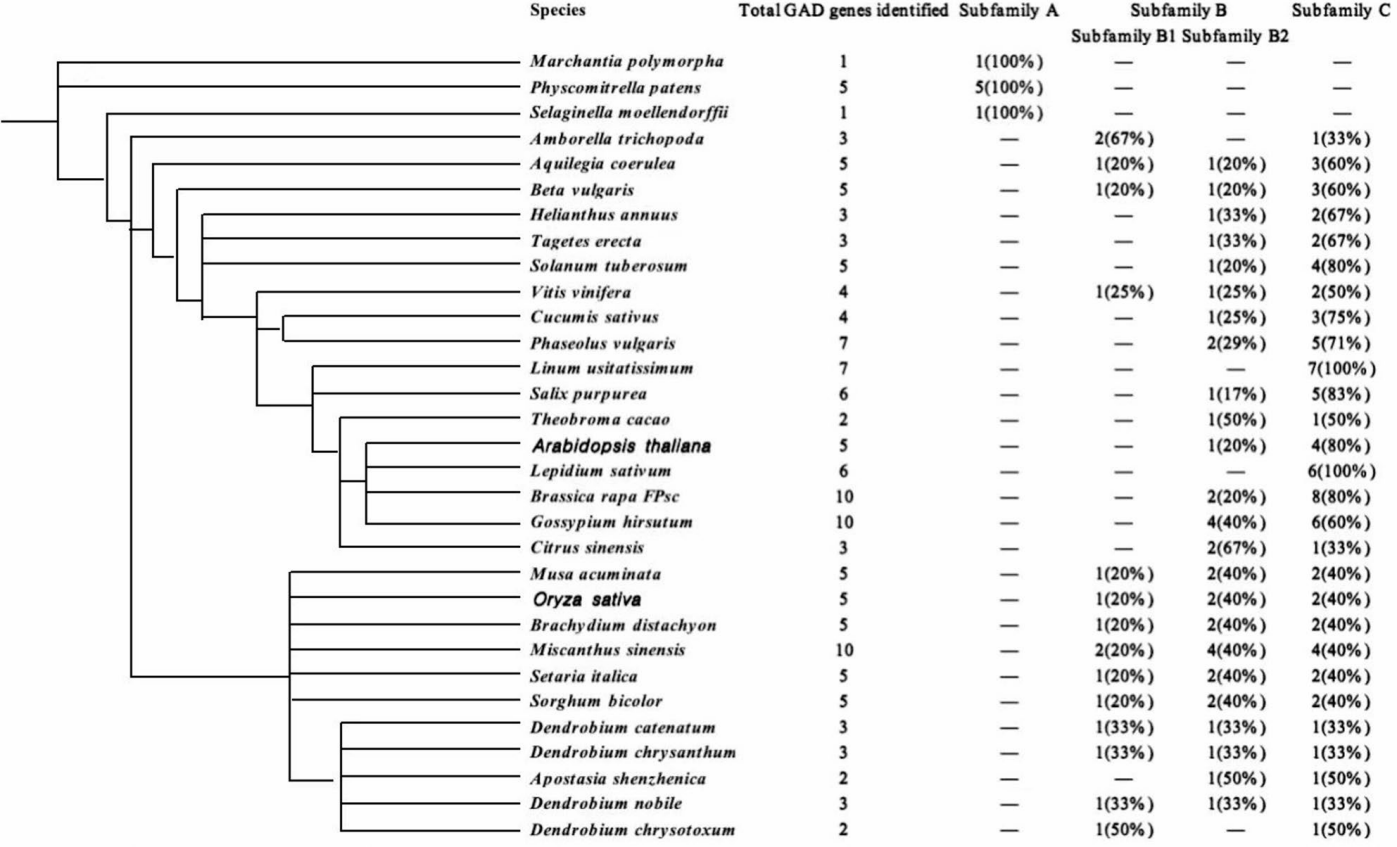
GAD genes identified across 31 plant species. The number of GAD genes detected in each sequenced genome is shown, with percentages indicated in parentheses. A dash (“-”) denotes the absence of GAD genes in the corresponding subfamily

A total of 182 GAD protein sequences were identified across 31 out of the 35 selected species, including *Arabidopsis* and rice. Notably, GAD sequences were not detected in *Porphyra umbilicalis*, *Chlamydomonas reinhardtii*, *Dunaliella salina*, and *Micromonas strain RCC299*, suggesting that the evolution of plant GAD genes may have originated after the divergence of Embryophyta. The number of GAD genes varied across species (Fig. 1). For example, *Selaginella* and *Marchantia polymorpha* contained only one GAD gene; cacao, *D. chrysotoxum* and *A. shenzhenica* each had two, and the remaining species had more.

**Fig. 2:**
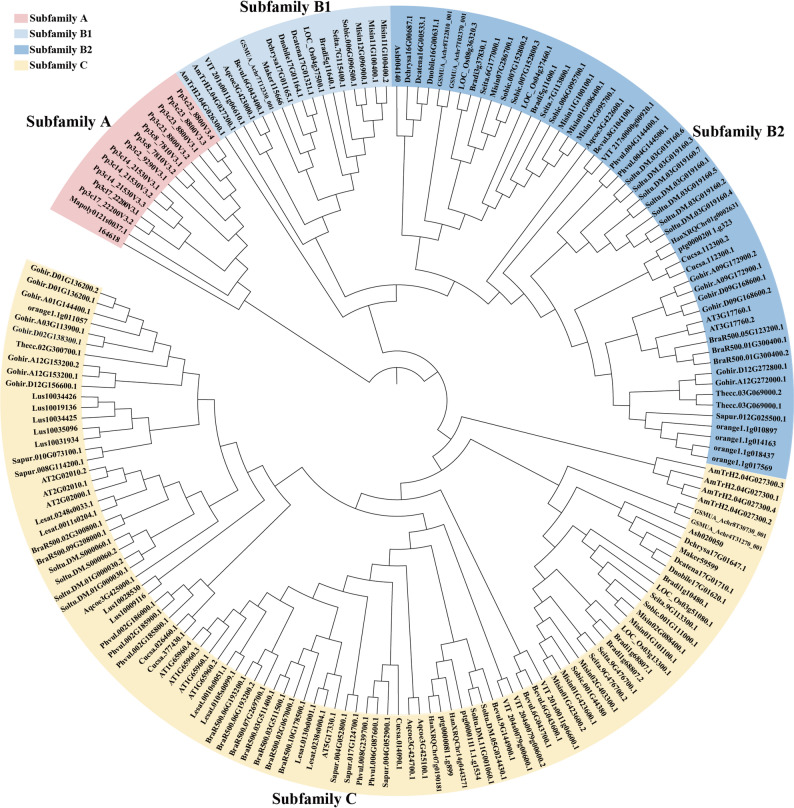
Phylogenetic tree of 182 GAD proteins from 31 plant species constructed using the Maximum Likelihood (ML) method implemented in PhyML 3.0. The tree reveals three major phylogenetic subfamilies (A, B, and C)

To elucidate the evolutionary relationships among plant GAD proteins, a maximum likelihood phylogenetic tree was constructed using the identified sequences (Fig. 2). Based on sequence similarity, tree topology, gene structures, and conserved motif compositions, the GAD gene family was classified into three major subfamilies: subfamilies A, B, and C. Subfamily C contained the largest number of members (98 GADs), followed by subfamily B (70 GADs), and subfamily A (14 GADs). Furthermore, subfamily B was subdivided into two distinct clades: B1 (17 GADs) and B2 (53 GADs). The number of GADs per species and their subfamily classifications were presented in Supplementary 1 Table S6. And the species in this table are arranged according to their phylogenetic relationships mainly according to Phytozome (Fig. 1).

The distribution of GAD members across subfamilies showed distinct evolutionary patterns:

1. Subfamily A was absent in angiosperms and included only members from basal Embryophytes such as *Selaginella moellendorffii*, *Marchantia polymorpha*, and *Physcomitrella patens*, indicating an ancient evolutionary lineage.

The distribution pattern of GADs among subfamilies B1, B2, and C appeared to correlate with the monocot–dicot divergence. Most monocot species, including banana, *Brachypodium distachyon*, *Miscanthus sinensis*, *foxtail millet*, *sorghum*, and several *Dendrobium* species, possessed members in subfamilies B1, B2, and C, showing a relatively balanced distribution across these groups. In contrast, GADs in dicots were predominantly concentrated in subfamilies B2 and C. Dicot species in subfamilies B2 and C included common bean, potato, sunflower, marigold, cucumber, cacao, *Salix purpurea*, *Arabidopsis*, *Brassica rapa*, sweet orange, and cotton.

Only two species, *A. trichopoda* and *Dendrobium chrysotoxum Lindl*, contained GAD genes in both subfamilies B1 and C. Additionally, *Linum usitatissimum* (flax) and *Lepidium sativum* (garden cress) possessed GAD genes exclusively in subfamily C.

Interestingly, *A. trichopoda* showed basal positioning in subfamilies B, B1, and C, highlighting its phylogenetic significance. In subfamilies B1, B2, and C, GAD genes from monocot and dicot species generally clustered into distinct clades.

(2)Subcellular localization analysis revealed that GAD proteins localization becomes more diverse with evolution, and they were predominantly localized in the cytoplasm (122 genes), followed by the chloroplast (34 genes), cytoskeleton (16 genes), and plasma membrane (1 gene) (Supplementary Table S5). Additionally, cytoskeleton-localized GADs were mainly identified in dicot species, and several GAD proteins showed multiple subcellular localizations, including combinations with the mitochondria, nucleus, or chloroplast.

- The physicochemical property analysis indicated that most GAD proteins in both monocot and dicot species had instability indices exceeding 40, a threshold commonly associated with protein instability (Supplementary Table S5). In contrast, GAD proteins from *M. polymorpha*, *P. patens*, *Selaginella*, *Amborella*, sunflower, marigold, potato, grapevine, and garden cress had notably lower instability indices, suggesting higher protein stability in these species.
- The predicted isoelectric points (pI) of GAD proteins ranged from 4.44 (*Lus10031934*) to 9.93 (*Gohir.D09G168600.2*). Protein lengths varied widely, from 89 amino acids (*PAC_32933239*) to 641 amino acids (*Lus10035096*), with most sequences clustering around 500 amino acids in length.

### Chromosomal localization of GAD genes

**Fig. 3:**
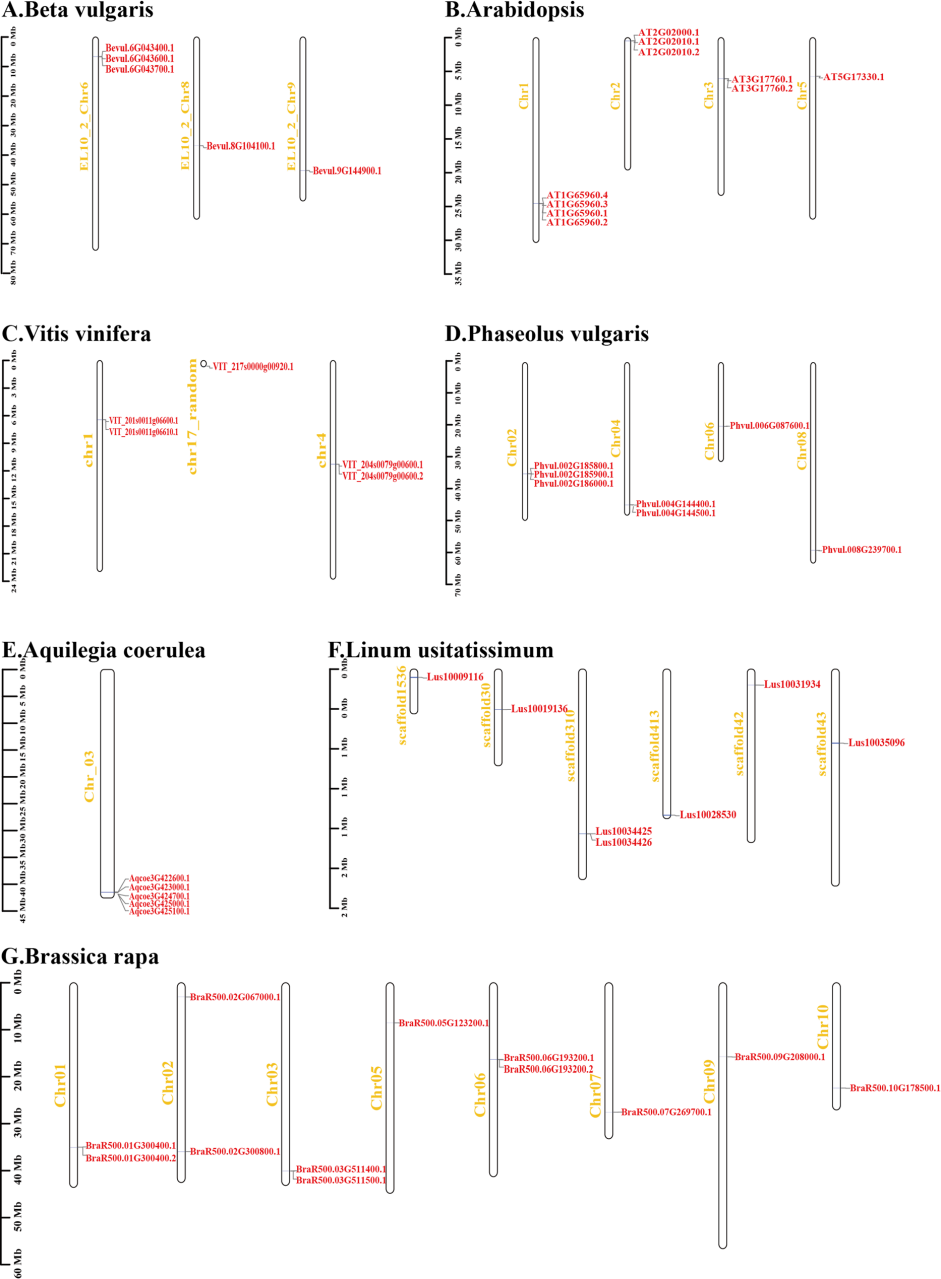
Chromosome distribution of GAD genes in sugar beet (**A**), *Arabidopsis* (**B**), grapevine (**C**), common bean (**D**), *Aquilegia coerulea* (**E**), flaxseed (**F**), and *Brassica rapa* (**G**). The scale of the genome size was given on the left

**Fig. 4:**
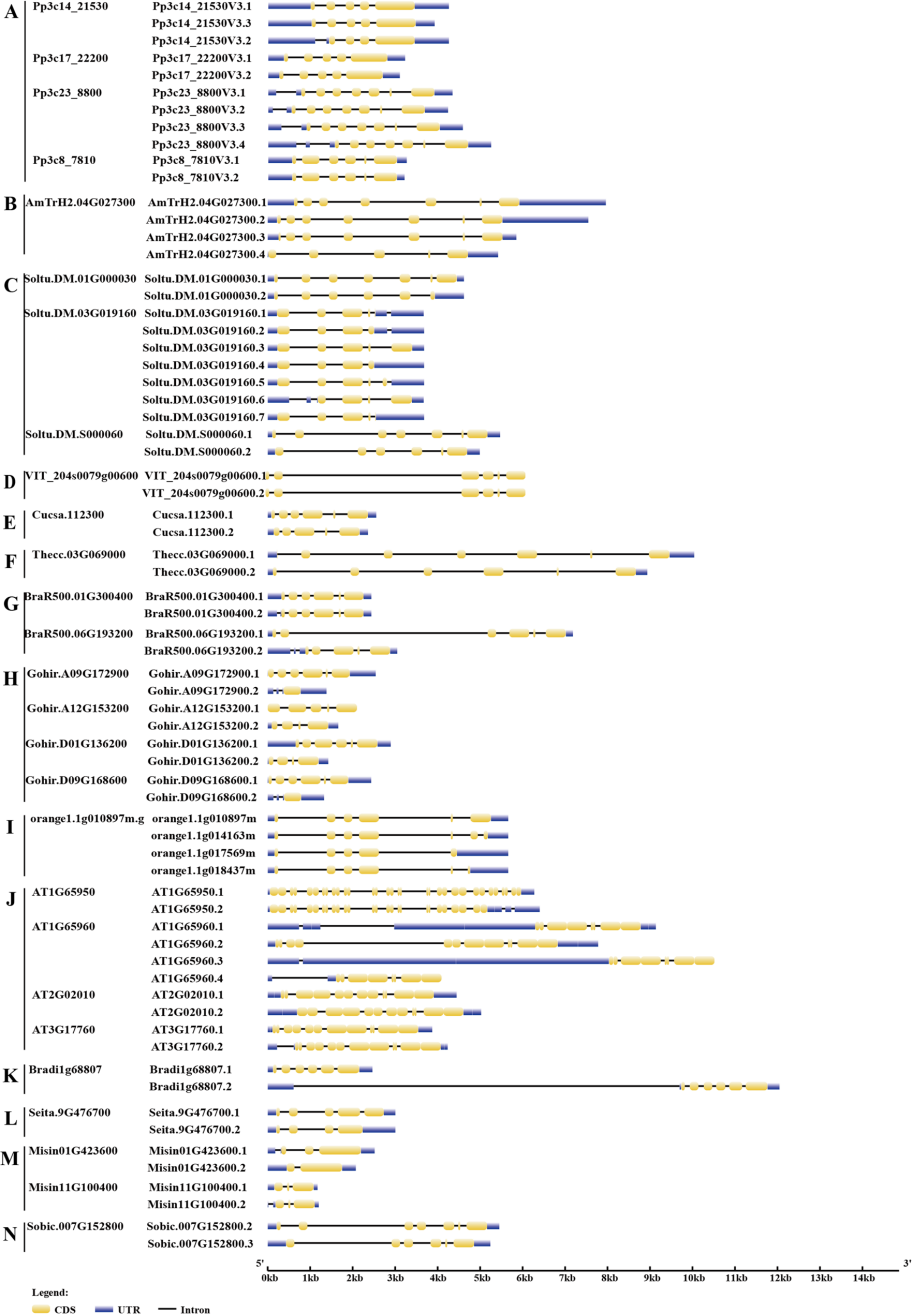
Annotated alternative splicing (AS) events of GAD genes in *Physcomitrella patens* (**A**), *Amborellaceae* (**B**), potato (**C**), grapevine (**D**), cucumber (**E**), cacao (**F**), Brassica rapa(**G**), cotton (**H**), orange (**I**), *Arabidopsis* (**J**), *Brachypodium distachyon* (K), foxtail millet (**L**), *Miscanthus sinensis* (**M**), sorghum (**N**). The scale of the gene size was given at the bottom

Chromosomal localization provides important insights into the evolutionary history and functional diversification of gene families. Our analysis revealed that GAD genes are unevenly distributed across chromosomes, with most located near telomeric regions. Tandemly duplicated GAD genes were predominantly identified in dicot species, including *Amborella trichopoda*, *Aquilegia coerulea*, *Beta vulgaris*, *Vitis vinifera*, *Phaseolus vulgaris*, *Salix purpurea*, *Brassica rapa*, and *Arabidopsis thaliana.* The chromosomal distributions of several representative dicot species are illustrated in Fig. 3, and the classification of gene duplication types is summarized in Supplementary Table S4. Annotated alternative splicing (AS) events were detected in approximately half of the examined species (Fig. 4), including *Physcomitrella patens*, *Amborella trichopoda*, potato, grapevine, cucumber, *Arabidopsis*, cacao, *Brassica rapa*, cotton, *Citrus sinensis*, *Brachypodium distachyon*, *Miscanthus sinensis*, foxtail millet, and sorghum species.

Details of tandem duplications and AS events are summarized below:

1. In *Physcomitrella patens*, five GAD genes were identified, with AS (Fig. 4A) observed in four of them: *Pp3c8_7810(Pp3c8_7810V3.1–3.2.2)*,* Pp3c14_21530(Pp3c14_21530V3.1–3.3.3)*,* Pp3c17_22200(Pp3c17_22200V3.1–3.2.2)*,* Pp3c23_8800(Pp3c23_8800V3.1–3.4.4*).
2. In *Amborella trichopoda*, three GAD genes were detected. *AmTrH2.04G027300* exhibited four AS isoforms (Fig. 4B). A tandem pair (*AmTrH2.04G027200.1* and *AmTrH2.04G027300.1*) was identified on chromosome 4.
3. In *Aquilegia coerulea*, five GAD genes were detected, with a tandem gene pair (*Aqcoe3G425000* and *Aqcoe3G425100*) located on chromosome 3.
4. In *Beta vulgaris*, five GAD genes were identified, including a tandem pair (*Bevul.6G043600.1*, *Bevul.6G043700.1*)
5. In potato, five GAD genes were identified, with AS (Fig. 4C) in: *Soltu.DM.01G000030* (2 isoforms), *Soltu.DM.03G019160* (7 isoforms), *Soltu.DM.S000060* (2 isoforms).
6. In grapevine, four GAD genes were found. *VIT_204s0079g00600* had two isoforms (Fig. 4D), and a tandem pair (*VIT_201s0011g06600.1* and *VIT_201s0011g06610.1*) was identified on chromosome 1.
7. In cucumber, four GAD genes were identified, with AS in *Cucsa.112300* (2 isoforms) (Fig. 4E).
8. In common bean, seven GAD genes were identified. Among them, *Phvul.004G144400.1* was classified as a WGD/segmental duplicate, while *Phvul.004G144500.1* was identified as a tandem duplicate on chromosome 4. In addition, *Phvul.002G185800*, *Phvul.002G185900*, and *Phvul.002G186000* formed a tandem duplication cluster on chromosome 2.
9. In *Salix purpurea*, six GAD genes were identified, with a tandem pair on chromosome 4 (*Sapur.004G052800* and *Sapur.004G052900*).
10. In *cacao*, two GAD genes were found, with AS in *Thecc.03G069000* (2 isoforms) (Fig. 4F).
11. In *Brassica rapa*, ten GAD genes were detected, with AS (Fig. 4G) in: *BraR500.01G300400* (2 isoforms), *BraR500.06G193200* (2 isoforms). *BraR500.03G511400.1* was classified as a WGD/segmental duplicate, while *BraR500.03G511500.1* was identified as a tandem duplicate on chromosome 3.
12. In cotton, ten GAD genes were detected, AS (Fig. 4H) was observed in several genes, including *Gohir.A09G172900*, *Gohir.A12G153200*, *Gohir.D01G136200*, and *Gohir.D09G168600* (2 isoforms each).
13. In sweet orange, three GAD genes were detected, with multiple isoforms (Fig. 4I) from *orange1.1g010897* (*orange1.1g014163*/*orange1.1g017569*/*orange1.1g018437*).
14. In *Arabidopsis*, AS (Fig. 4J) was observed in *AT1G65960* (4 isoforms), *AT2G02010* (2 isoforms), and *AT3G17760* (2 isoforms). *AT2G02000* was classified as a WGD/segmental duplicate, while *AT2G02010* was identified as a tandem duplicate on chromosome 2.
15. Five GAD genes were found in *Brachypodium distachyon*, with AS (Fig. 4K) in *Bradi1g68807* (2 isoforms).
16. Five GAD genes were present in foxtail millet, with AS (Fig. 4L) observed in *Seita.9G476700* (2 isoforms).
17. In *Miscanthus sinensis*, ten GAD genes were found, with AS (Fig. 4M) in *Misin01G423600* and *Misin11G100400* (2 isoforms each).
18. In sorghum, five GAD genes were identified, including *Sobic.007G152800*, which exhibited AS (2 isoforms) (Fig. 4N).

These findings indicate the potential presence of both tandem duplication and alternative splicing within the GAD gene family, supporting their roles in the functional diversification and adaptive evolution of GADs across plant lineages.

### Protein conserved Motifs, domain architecture and Exon-intron structure analysis

**Fig. 5:**
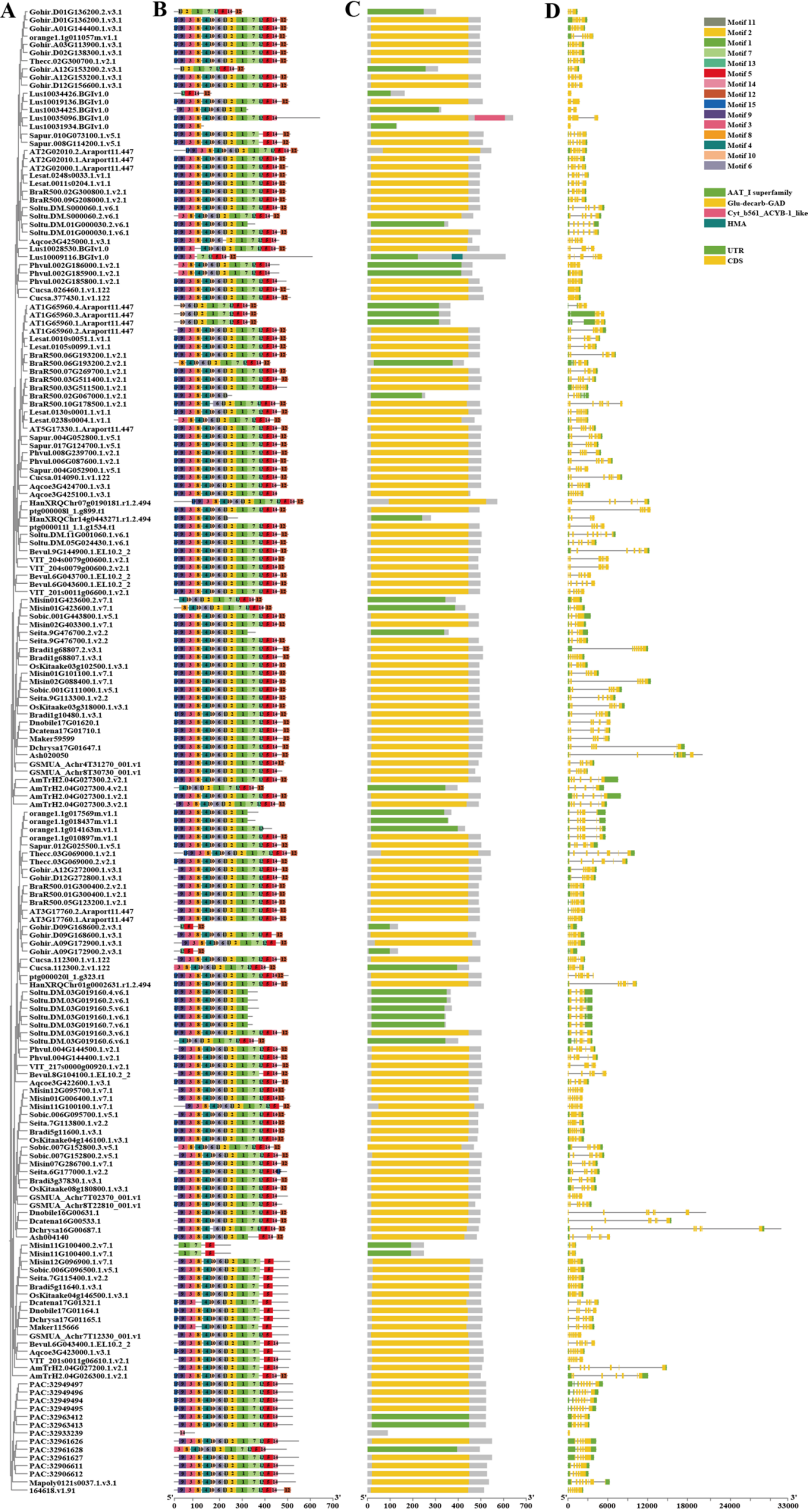
Conserved motifs, protein domains, and exon-intron structures of GAD gene family. **A** Phylogenetic tree of GAD genes constructed using the Maximum Likelihood (ML) method in PhyML 3.0. **B** Conserved motifs architecture of GAD proteins, identified using the MEME Suite and visualized with TBtools. **C** Conserved protein domains of GAD family members. (D)Exon–intron organization of GAD genes, illustrating gene structural diversity among family members

To gain insights into the structural features of the GAD gene family, we conducted a comprehensive analysis of conserved motifs (Fig. 5B), protein domain architectures (Fig. 5C), and exon–intron structures (Fig. 5D).

1. Conserved Motif Analysis:
  The GAD proteins exhibit a highly conserved motif arrangement across most members, following the sequence: motif15–motif9–motif3–motif8–motif4–motif10–motif6–motif11–motif2–motif1–motif7–motif13–motif5–motif14–motif12. While this motif pattern is largely conserved, certain sequences lack motifs at either the N- or C-terminal ends, potentially reflecting subfunctionalization or neofunctionalization during evolution. This conservation highlights the fundamental biochemical roles of GAD proteins across diverse plant species.
  In terms of motif number, subfamilies C and B2 showed a relatively consistent motif count across members, with minor deviations likely due to incomplete gene models or sequencing artifacts. This structural uniformity may reflect evolutionary constraints that maintain motif integrity within these subgroups.
  Subfamily-specific motif distributions were also observed. Motif12, predominantly located at the C-terminal region, is mainly present in subfamilies B2 and C, but is rare in subfamilies A and B1. Similarly, motif13, which is also located near the C-terminus, is widely distributed in subfamilies A, B2, and C, but is rare absent in B1. Given that protein motifs often reflect distinct evolutionary histories and biochemical functions, such patterns may suggest functional divergence among subfamilies.
2. Domain Architecture Analysis:
  Most GAD proteins belong to the pyridoxal phosphate (PLP)-dependent glutamate decarboxylase (GAD) family, characterized by a conserved PLP-binding domain. A few members were classified within the aspartate aminotransferase (AAT) superfamily (fold type I) of PLP-dependent enzymes. Notably, unique domain architectures were identified in *Linum usitatissimum* (flaxseed), including a heavy metal-associated (HMA) (Lus10009116) domain and a plant cytochrome b561 domain (which encompasses the carbon monoxide oxygenase ACYB-1 domain)(Lus10035096) in C-terminal regions. These domain variations may imply additional functional diversification in specific lineages. Interestingly, although motif12 is prominently present in subfamilies B2 and C, its biological significance remains unclear and may not contribute directly to core GAD enzymatic function.
3. Exon-Intron Structure Analysis:
  The number of coding sequences (CDSs) among GAD genes ranges from one to eight. For example, *Pp3c8_9290V3.1* contains only a single CDS, while *AT5G17330.1* comprises eight CDS regions. Most GAD genes, however, possess six or seven CDS regions, suggesting a conserved exon–intron architecture across subfamilies and species.
  GAD genes with similar intron lengths often cluster within the same phylogenetic sub-branches, suggesting shared evolutionary origins. Notably, some genes, such as ptg000008l_1.g899, harbor exceptionally long introns—for instance, its second intron spans approximately 10,458 bp. Interestingly, GAD genes in orchid species exhibited relatively longer introns compared to those in other taxa, especially within subfamily B2. This may reflect lineage-specific genomic expansion or intron retention events during evolution.

### Cis-acting element analysis of the 2000 bp upstream promoter and intron regions

To further explore the potential regulatory mechanisms of GAD genes, we analyzed cis-acting elements located within the 2000 bp upstream promoter regions of each gene (Supplementary Table S3). In addition to core promoter elements such as the transcription start site, TATA box, and CAAT box, the analysis revealed a predominance of light-responsive elements across nearly all GAD promoters, with copy numbers ranging from 1 to 22 (Supplementary 5, Figure S1). Notably, sweet orange exhibited the highest abundance of these elements, suggesting that light may play a prominent role in regulating GAD gene expression in this species.

**Fig. 6:**
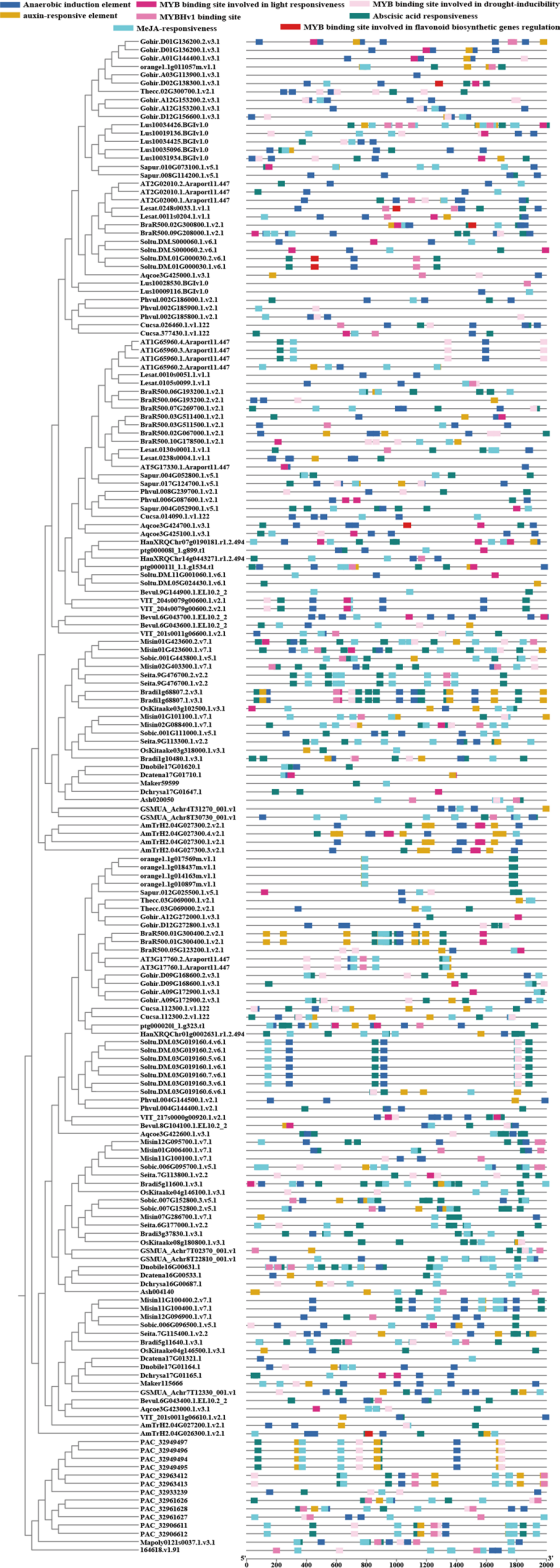
Cis-acting elements identified in the promoter regions of GAD gene family members. This figure presents the distribution of key cis-regulatory elements involved in hormonal and stress responses, including methyl jasmonate (MeJA)-responsive elements, abscisic acid (ABA)-responsive elements, anaerobic induction elements (ARE), MYB binding sites, MYBHv1 binding sites, and auxin-responsive elements

In addition to light-responsive elements, we identified several other cis-acting elements. Elements responsive to methyl jasmonate (MeJA), abscisic acid (ABA), and anaerobic induction were prevalent. Furthermore, various MYB binding sites were detected, including those involved in light responsiveness, flavonoid biosynthesis regulation, and drought inducibility (Fig. 6).

Other cis-acting elements identified include those responsive to auxin, salicylic acid, and gibberellins, as well as elements associated with low-temperature responsiveness, meristem expression, wound responsiveness, and defense-related signaling. Elements involved in zein metabolism regulation, AT-rich DNA binding protein (ATBP-1) binding, and endosperm-specific expression were also detected, albeit at lower frequencies. In addition, a few elements related to circadian regulation and seed-specific gene expression were found.

The wide variety of cis-acting regulatory elements suggests that GAD genes are subject to multifaceted regulation and may be involved in diverse biological processes, including growth, development, metabolic regulation, and adaptive responses to environmental stimuli.

**Fig. 7:**
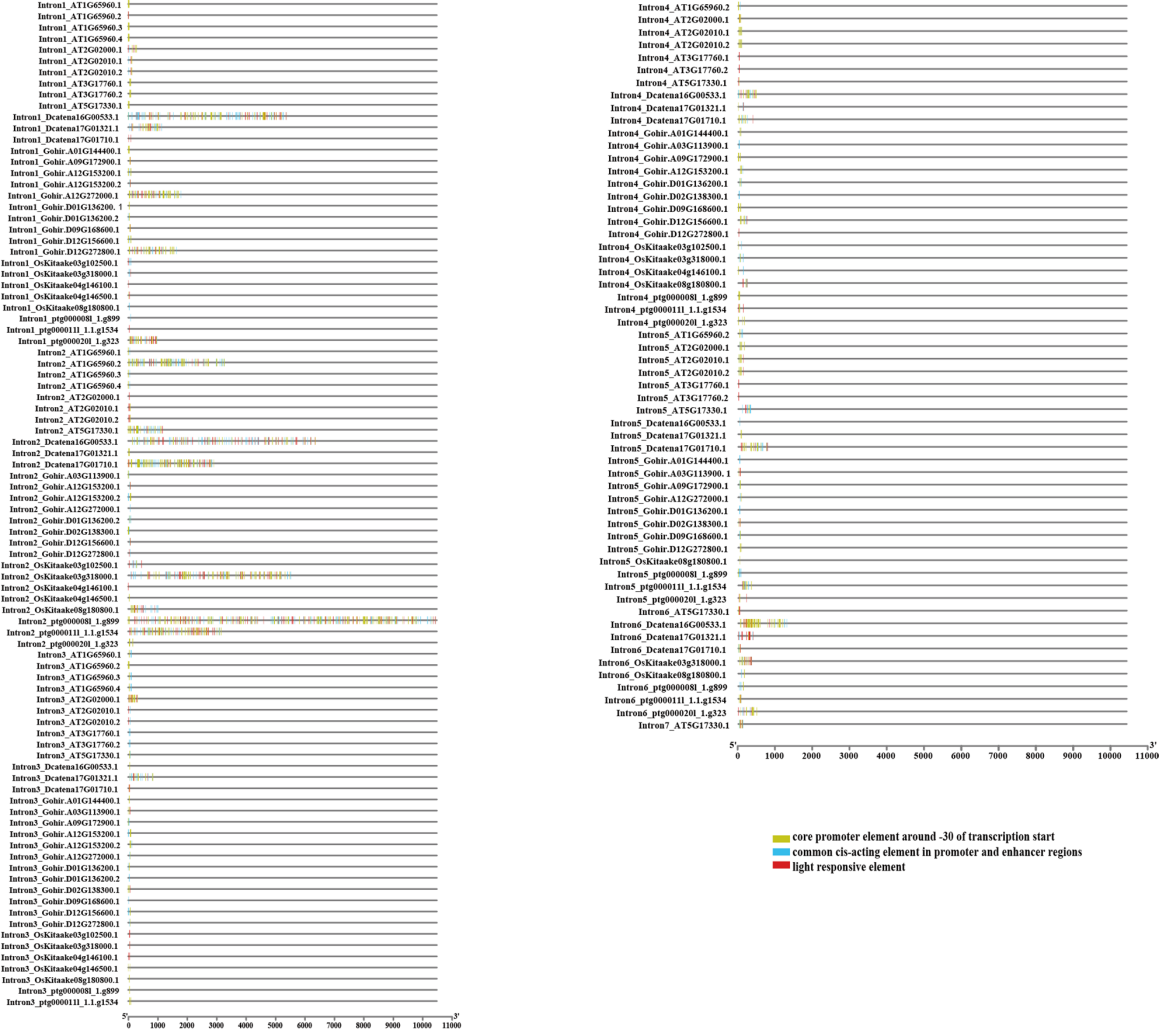
Cis-acting regulatory elements identified within introns of GAD gene family members in *Arabidopsis thaliana*, *Dendrobium catenatum*, cotton (*Gossypium spp*.), rice (*Oryza sativa*), and marigold (*Tagetes spp.*). This figure illustrates the presence and distribution of intronic cis-acting elements potentially involved in transcriptional regulation across five representative plant species.

Meanwhile, we also examined cis-acting regulatory elements located within intronic regions. Across most introns, we observed a predominance of transcription start sites, core promoter and enhancer regions, as well as light-responsive elements (Fig. 7).

In *D.catenatum*, introns were generally longer than those in other species. Notably, both the first and second introns of *Dcatena16G00533.1* harbored numerous cis-regulatory elements, followed by the second intron of *Dcatena17G01710.1*. In marigold, the second intron of *ptg000008l* was the longest among all examined GAD genes and harbored the highest number of regulatory elements. In *Arabidopsis*, the second intron of *AT1G65960.2* had the greatest regulatory element density, followed by the second intron of *AT5G17330.1*. Similarly, in rice, the second intron of *OsKitaake03g318000.1* harbored the most cis-acting elements, followed by the second intron of *OsKitaake08g180800.1*. While, in cotton, the first intron of *Gohir.A12G272000.1* showed the greatest abundance of transcription-related elements, followed by the first intron of *Gohir.D12G272800.1*. These results suggest that the first or second introns may play a key role in regulating GAD gene expression, as supported by corresponding RNA-seq expression profiles (see Sect. 3.5 for dataset details).

In addition to core regulatory elements, elements responsive to methyl jasmonate (MeJA), anaerobic conditions, abscisic acid (ABA) were prevalent, followed by gibberellins, auxin, salicylic acid (SA), low-temperature responsiveness, meristem expression, circadian control, and zein metabolism regulation and so on. MYB-binding sites were also detected, including those involved in light response, flavonoid biosynthesis, and drought inducibility. The presence of such a diverse array of regulatory elements within introns underscores their potential importance in fine-tuning GAD gene transcription in response to developmental cues and environmental stimuli.

### Expression pattern of GAD genes in different tissues development

**Fig. 8:**
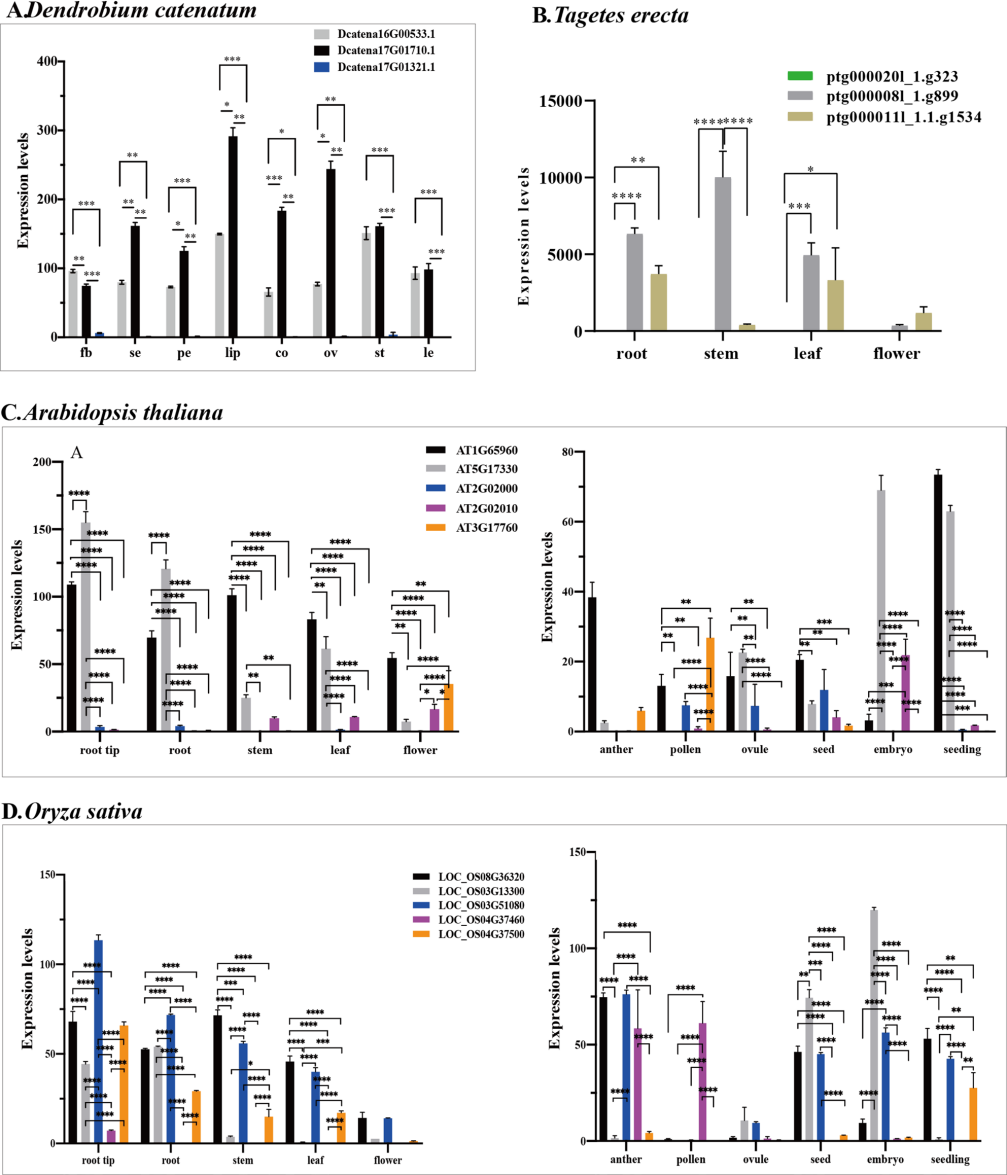
Expression comparison of GAD genes in *Dendrobium catenatum*, *Tagetes erecta*, *Arabidopsis thaliana*, and *Oryza sativa* based on RNA-seq data. **A**. Gene expression levels were quantified as log₂-transformed TPM values using TBtools software. The data represent mean expression values from three biological replicates for each gene. Tissue abbreviations: fb-flower bud, se-sepal, pe-petal, lip-lips, co-column, ov-ovary, st-stem, le-leaf. **B**. Gene expression levels were determined as log₂-transformed TPM values. The results represent mean values from five biological replicates. **C.** Gene expression levels were quantified as FPKM values. **D**. Gene expression levels were quantified as FPKM values. Error bars represent the standard error (SE) of the mean. Statistical significance was determined using one-way analysis of variance (ANOVA). Asterisks indicate significance: *p <.01, **p <.01, ***p <.001, ****p <.0001

**Fig. 9:**
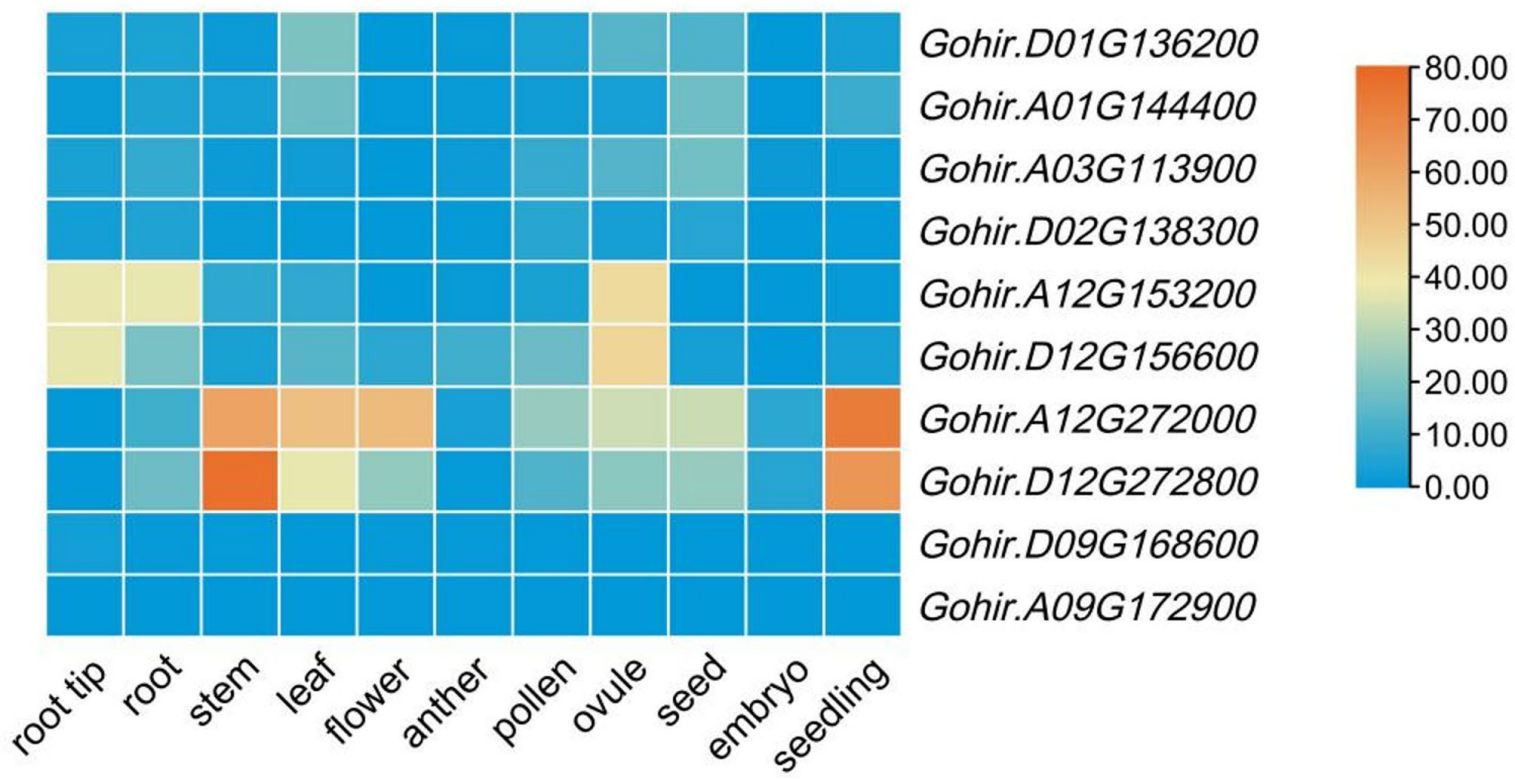
Heatmap of GAD gene expression in cotton across 11 tissues: root tip, root, stem, leaf, flower, anther, pollen, ovule, seed, embryo, and seedling. Gene expression levels were quantified as FPKM values. Expression levels are shown as a color scale from deep sky blue (low) to orange red (high), with the scale indicated on the right

To explore the specific expression dynamics of the GAD gene family, we analyzed RNA-seq data from *D. catenatum*(Fig. 8A), marigold(Fig. 8B), *Arabidopsis*(Fig. 8C), rice(Fig. 8D), and cotton (Fig. 9)across different organs.

In *D. catenatum*, *Dcatena17G01321.1* (subfamily B1) exhibited markedly lower expression levels compared to *Dcatena17G01710.1* (subfamily C) and *Dcatena16G00533.1* (subfamily B2) in both reproductive tissues (flower bud, sepal, petal, lip, column, ovary, and pollinia) and vegetative tissues (green root, white root, stem, and leaf). Notably, *Dcatena17G01710.1* showed the highest expression levels in reproductive organs such as sepals, petals, lips, columns, and ovaries, while in the flower bud, its expression was significantly lower than that of *Dcatena16G00533.1*. *Dcatena17G01710.1* and *Dcatena16G00533.1* showed similar expression levels in stems and leaves.

In marigold, *ptg000020l_1.g323* (subfamily B2) exhibited significantly lower expression than *ptg000008l_1.g899* (subfamily C) in the root, stem, and leaf. And it also had significantly lower expression than *ptg000011l_1.1.g1534* (subfamily C) in the root and leaf. Furthermore, *ptg000011l_1.1.g1534* exhibited significantly lower expression than *ptg000008l_1.g899* in the stem.

In *Arabidopsis*, several notable trends emerged:

1.  *AT3G17760* (subfamily B2) was generally weakly expressed across tissues, except for a marked increase in flowers and pollen, implying a potential role in floral organ development.
2.  *AT5G17330* (subfamily C) was expressed at significantly higher levels than all other members in the root tip, root, and embryo, and showed elevated expression in leaf, ovule, and seedling. However, it was relatively weakly expressed in flower, anther, and pollen. *AT1G65960* (subfamily C) exhibited significantly higher expression levels than *AT2G02000*, *AT2G02010*, and *AT3G17760* in most tissues, including root tip, root, stem, leaf, flower, and seedling. It also displayed relatively high expression across other tissues, excluding the embryo. Thus, *AT1G65960* and *AT5G17330* have similar expression pattern and might function in mostly tissues, except flower organs or the embryo.
3. *AT2G02000* (subfamily C) and *AT2G02010* (also subfamily C) exhibited low expression in most tissues. Interestingly, *AT2G02010* showed significantly higher expression in flower and embryo, suggesting possible tissue-specific functions in floral and embryonic development.

In rice, the following patterns were observed:

1.  *LOC_OS04G37500* (subfamily B1) was expressed in vegetative tissues but was largely absent in reproductive organs, implying a function confined to non-reproductive development.
2.  *LOC_OS08G36320* (subfamily B2) showed significantly higher expression than *LOC_OS03G13300/LOC_OS04G37460* in root tip, stem, leaf, and seedling, and was more highly expressed than *LOC_OS04G37500* in root, leaf, anther, seed, and seedling. It was significantly lower than *LOC_OS03G51080* in root tip, root, and embryo, and lower than *LOC_OS03G13300* in seed and embryo. This suggests *LOC_OS08G36320* plays broad roles in vegetative tissues but may be excluded from floral reproductive functions.
3.  *LOC_OS04G37460* (subfamily B2) was primarily expressed in anther and pollen, suggesting a specialized role in male gametogenesis. But low expression in most other tissues.
4.  *LOC_OS03G51080* (subfamily C) showed consistently high expression across most tissues except floral organs(i.e. flower, pollen, ovule).
5. *LOC_OS03G13300* (subfamily C) had high expression in root-related tissues and during early development stages, but low expression in reproductive tissues.

In cotton, heatmap analysis revealed five major findings:

1.  *Gohir.D09G168600* and *Gohir.A09G172900* (both subfamily B2) were minimally expressed across all tissues.
2.  *Gohir.D12G272800* and *Gohir.A12G272000* (both subfamily B2) were broadly expressed in both reproductive and vegetative tissues, indicating a more generalized function.
3.  *Gohir.D12G156600* and *Gohir.A12G153200* (both subfamily C) were expressed in most organs except seed, embryo, and seedling, suggesting roles in mature tissues.
4. *Gohir.D02G138300*, *Gohir.D01G136200*, *Gohir.A03G113900*, and *Gohir.A01G144400* (subfamily C) exhibited weak expression overall, implying minor or specialized functions.

## Discussion

### Evolutionary diversification

GAD genes have been extensively studied across microorganisms, plants, and animals. In microorganisms and animals, typically one or two isoforms of GAD exist, with some microorganisms possessing only a single GAD gene [38, 39]. In contrast, the number of GAD genes in plants ranges from one to more than ten, commonly exceeding two. This expansion, consistent with previous reports [10, 34], suggests both functional diversification and amplification in plants, underlining the evolutionary complexity of the gene family and its potential for broader biological roles. The GAD genes identified in cotton were consistent with recent findings [30], indicating high accuracy and reliability in gene identification of the present study.

Phylogenetic analysis suggests that plant GADs may have originated in early Embryophyta. Notably, subfamily A was absent in angiosperms, indicating that it may constitute an ancient evolutionary lineage. The distribution pattern of GADs further implies that monocot diversification occurred earlier with limited subsequent expansion, whereas dicot GADs likely underwent later divergence and expansion events, primarily within subfamilies B2 and C. From a structural perspective, GADs 3D structure preliminary explored by AlphaFold displayed a high degree of similarity, supporting the conserved nature of GAD proteins across species(Representative images see Supplementary 7).

Furthermore, the GAD genes of *A. trichopoda* were located at the base of subfamilies B, B1, and C, consistent with its status as a basal angiosperm lineage [56, 57]. Similarly, *Aquilegia coerulea* GADs were positioned near the base of subfamilies B1, B2, and C in the eudicot branch, aligning with its classification as a basal eudicot [58]. Additional basal representatives, such as grapevine and sugar beet (lower eudicots), and banana and orchids (early monocots), support this pattern. Collectively, these observations indicate that GAD genes could serve as valuable molecular markers for studying plant evolutionary history.

Segmental duplications, tandem duplications, and transposition events are widely recognized as critical drivers of gene family expansion in plants [59]. In our study, tandem duplication appears to be the predominant mechanism underlying the expansion of the GAD gene family in dicot species. Moreover, the observation of multiple annotated isoforms in many GAD genes suggests that alternative splicing may play a role in modulating their expression and functional diversity. Nevertheless, annotation alone cannot fully confirm these events. Future studies integrating isoform-level expression data will be necessary to validate and clarify the biological significance of alternative splicing within the GAD family. In contrast, lineage-specific gene loss might also happen in some species during evolution, for example, *A. trichopoda* and *Dendrobium chrysotoxum* GAD genes in subfamilies B1 and C, and flax and garden cress GAD genes just in subfamily C.

### Expression patterns and regulation

We observed that certain GAD genes, such as *Dcatena17G01321.1* in *D.catenatum*, *ptg000020l_1.g323* in marigold, *LOC_Os04g37500* in rice, *AT3G17760.1* in Arabidopsis, and *Gohir.D09G168600*/*Gohir.A09G172900* in cotton, exhibited consistently low expression levels across a range of tissues. Notably, these genes are located near the basal positions of their respective phylogenetic clades, suggesting that ancestral GAD genes may have been functionally replaced by more recently evolved paralogs. Nevertheless, these ancestral genes may still play essential roles in maintaining genetic diversity and preserving evolutionary continuity with early-diverging lineages.

Moreover, we found that phylogenetic proximity was often associated with similar expression profiles. For instance, *Dcatena17G01710.1* (subfamily C) and Dcatena16G00533.1 (subfamily B2), *ptg000008l_1.g899* (subfamily C) and *ptg000011l_1.1.g1534* (subfamily C), *AT1G65960* (subfamily C) and *AT5G17330* (subfamily C), *Gohir.D09G168600* and *Gohir.A09G172900* (both subfamily B2), *Gohir.D12G272800* and *Gohir.A12G272000* (both subfamily B2), *Gohir.D12G156600* and *Gohir.A12G153200* (both subfamily C), as well as *Gohir.D02G138300*,* Gohir.D01G136200*,* Gohir.A03G113900* and *Gohir.A01G144400* (all subfamily C) displayed highly similar expression patterns across multiple tissues, implying a conserved transcriptional regulation within subfamilies.

Overall, GAD gene expression showed tissue- and species-specific patterns. For example, in *D. catenatum*, both *Dcatena17G01710.1* (subfamily C) and *Dcatena16G00533.1* (subfamily B2) showed relatively high expression levels across nearly all the tissues, whereas *Dcatena17G01321.1* displayed markedly low expression. Similar but not identical trends were observed in marigold, indicating both conserved and lineage-specific regulatory mechanisms. In *Arabidopsis*, *AT1G65960* (GAD2) was expressed in nearly all tissues, consistent with previous findings [60]. Interestingly, *AT5G17330* (GAD1), previously reported to be root-specific (Zik et al., 1998), was also expressed in stem, leaf, flower, ovule, seed, embryo, and seedling tissues, suggesting a broader functional role. Furthermore, *AT3G17760* was mainly expressed in reproductive organs, indicating a potential function in floral organ development rather than vegetative tissues. A similar expression pattern was observed for *LOC_OS04G37460* in rice, which may also be involved in reproductive processes such as pollination.

Additionally, Previous studies have demonstrated that long introns can facilitate exon recombination, encode regulatory RNAs, and enhance gene expression through the inclusion of enhancers and other cis-regulatory elements [61–63]. Genes such as *Dcatena17G01710.1* and Dcatena16G00533.1 in *D. catenatum*, *ptg000008l_1.g899* and *ptg000011l_1.1.g1534* in marigold, AT1G65960 and *AT5G17330 in Arabidopsis*,* LOC_Os03g51080* and *LOC_Os08g36320* in rice exhibited high expression across multiple tissues. Structural analysis revealed that these genes possess significantly longer second introns. Similarly, *Gohir.A12G272000* and *Gohir.D12G272800* in cotton, which contain longer first introns, also exhibited high expression levels, consistent with prior observations in cotton [30]. Our cis-acting element analysis further confirmed that the first or second introns of these highly expressed GAD genes contained a greater number of promoter- and enhancer-like elements. In contrast, several GAD genes with shorter first or second introns consistently exhibited low expression across tissues, such as *Dcatena17G01321.1* in *D.catenatum*, *ptg000020l_1.g323* in marigold, *LOC_Os04g37500.1* in rice, *AT3G17760.1/AT2G02000* in Arabidopsis, and *Gohir.D09G168600/Gohir.A09G172900* in cotton. These results suggest a potentially generalizable relationship between intron length, cis-regulatory element density, and gene expression levels across plant species. However, as this remains a correlative observation, further experimental validation (e.g., reporter assays) would be needed to confirm a causal regulatory role.

Beyond the established roles of promoters and enhancers, introns can regulate gene expression via intron-mediated enhancement (IME) [64]. In certain cases, IME exerts a stronger influence on transcript abundance and expression pattern than promoters themselves [65–68]. Several conserved motifs responsible for IME have been defined in previous studies in *Arabidopsis* and rice, for example, “TTNGATYTG”, “CGATT”, “ARATCGA”, “GATTCG”, “KCGAGAR”, “TTTCGA”, “ACYCYRA”, and “GATCTGT” [66, 69–71]. These motifs are thought to be evolutionarily conserved across plant species [71–73] and are typically located near the promoter regions, particularly within the first intron [74]. To further investigate the potential regulatory role of IME in GAD gene expression, we screened the introns of GAD genes in *D. catenatum*, marigold, *Arabidopsis*, rice, and cotton for the presence of the eight known IME-related motifs(see Supplementary 1, Table S5). These motifs were variably detected across species, with a higher frequency in the first or second introns. Notably, genes with high expression levels generally exhibited a greater abundance of IME motifs, supporting the hypothesis that intron-mediated enhancement contributes to the regulation of GAD gene expression.

### Functional implications

Consistent with previous studies, our analysis of motifs and domain architecture revealed the highly conserved nature of GAD family proteins [30, 36]. And evolutionary variations were primarily observed in the N-terminal and C-terminal regions, which may contribute to the functional diversification of GAD genes [30]. Notably, in flax, we identified the presence of a heavy-metal-associated (HMA) domain and a plant-specific cytochrome b561 domain within the C-terminal region, suggesting a potential functional link between GAD proteins and redox activity or heavy metal detoxification. And the GAD HMA domain might contribute to its phytoremediation and the reduction of the metal content of soil [75].

In addition, subcellular localization analysis indicated that GAD proteins are predominantly localized in the cytoplasm. However, with increasing evolutionary divergence, their subcellular distribution appears to have become more diverse, potentially reflecting functional specialization. Collectively, these findings imply that further functional diversification of GAD proteins may emerge with ongoing plant evolution.

Cis-acting elements in GAD promoters further illuminate their functional diversity. These regulatory sequences mediate gene expression in response to developmental, physiological, and environmental cues [76, 77]. Our analysis revealed that the most abundant cis-acting elements were related to light responsiveness, followed by abscisic acid (ABA) response, methyl jasmonate (MeJA) responsiveness, anaerobic induction (ARE), and MYB binding sites. ABA and MeJA are key hormones that mediate stress responses, while ARE is typically associated with hypoxia-induced expression [26]; Mei et al., 2016; Huang et al., [30]. The high abundance of light-responsive and MYB-related elements suggests that GAD genes may play important roles in growth, metabolism, and stress adaptation.

## Conclusions

This study provides the most comprehensive evolutionary and expression analysis of the GAD gene family to date, revealing its complex diversification patterns across major plant lineages. Subfamily-specific expansion via tandem duplication and regulatory fine-tuning through intron length and cis-acting element composition suggest a dynamic interplay between genome architecture and transcriptional control. The correlation between intronic features and expression profiles reinforces the importance of non-coding regulatory regions in plant gene expression evolution. These findings not only shed light on the GAD gene family’s evolution but also offer valuable genomic resources for breeding stress-tolerant and developmentally optimized plant varieties.

## Supplementary Information

Supplementary Material 1.

Supplementary Material 2.

Supplementary Material 3.

Supplementary Material 4.

Supplementary Material 5.

Supplementary Material 6.

Supplementary Material 7.

## Data Availability

The datasets used and analyzed during the study are available from the corresponding author on reasonable request.
