# Supplementary figures and images for: Evolutionary dynamics and transcriptional diversification of GAD gene family in plants: a pan-species perspective in silico analysis
Source: BMC Plant Biol. 2025 Nov 22;25:1746. doi: 10.1186/s12870-025-07691-4 (PMC12750563; doi:10.1186/s12870-025-07691-4)

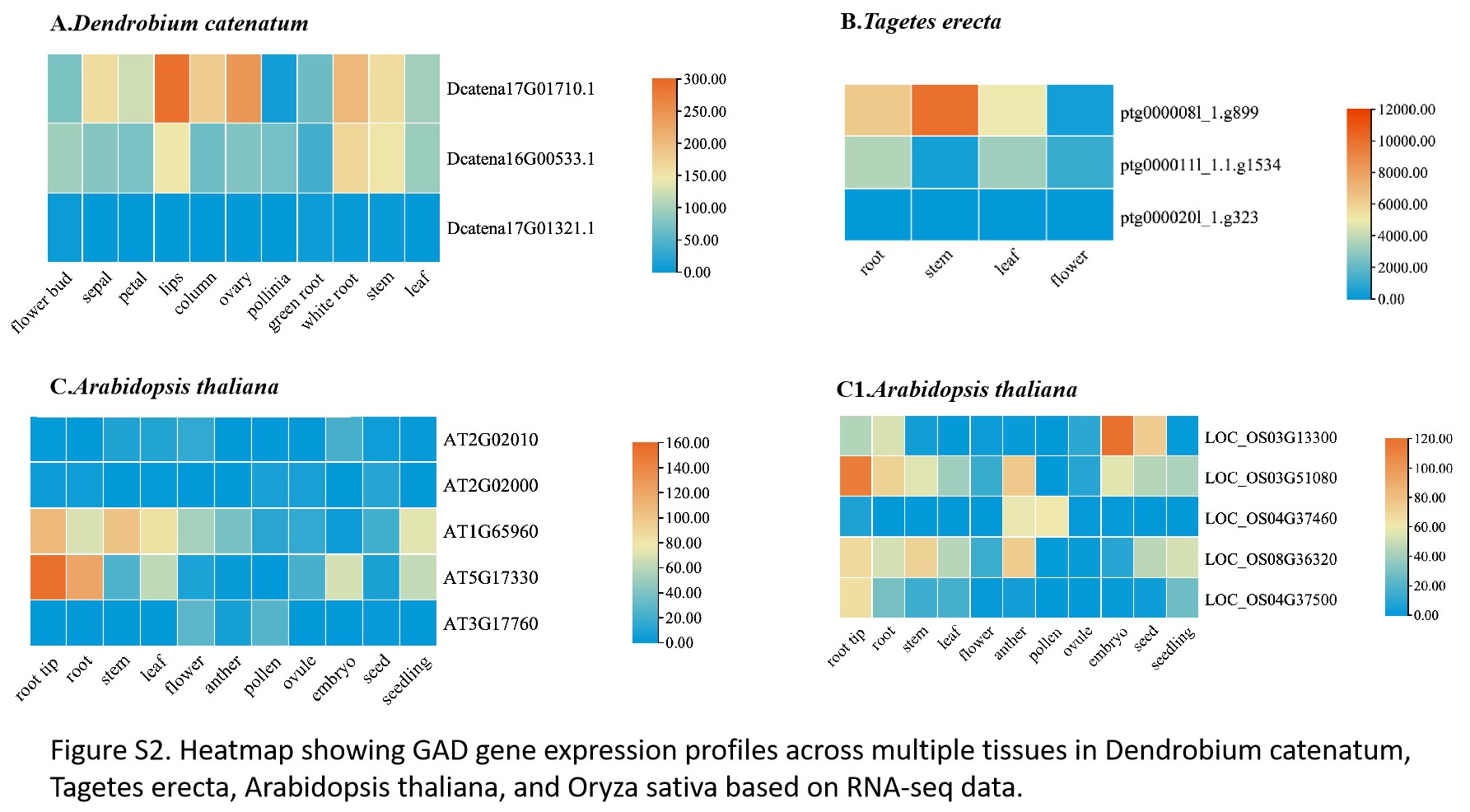

Supplement: Supplementary file 4 — Supplementary Material 4. [file 12870_2025_7691_MOESM4_ESM.jpg]

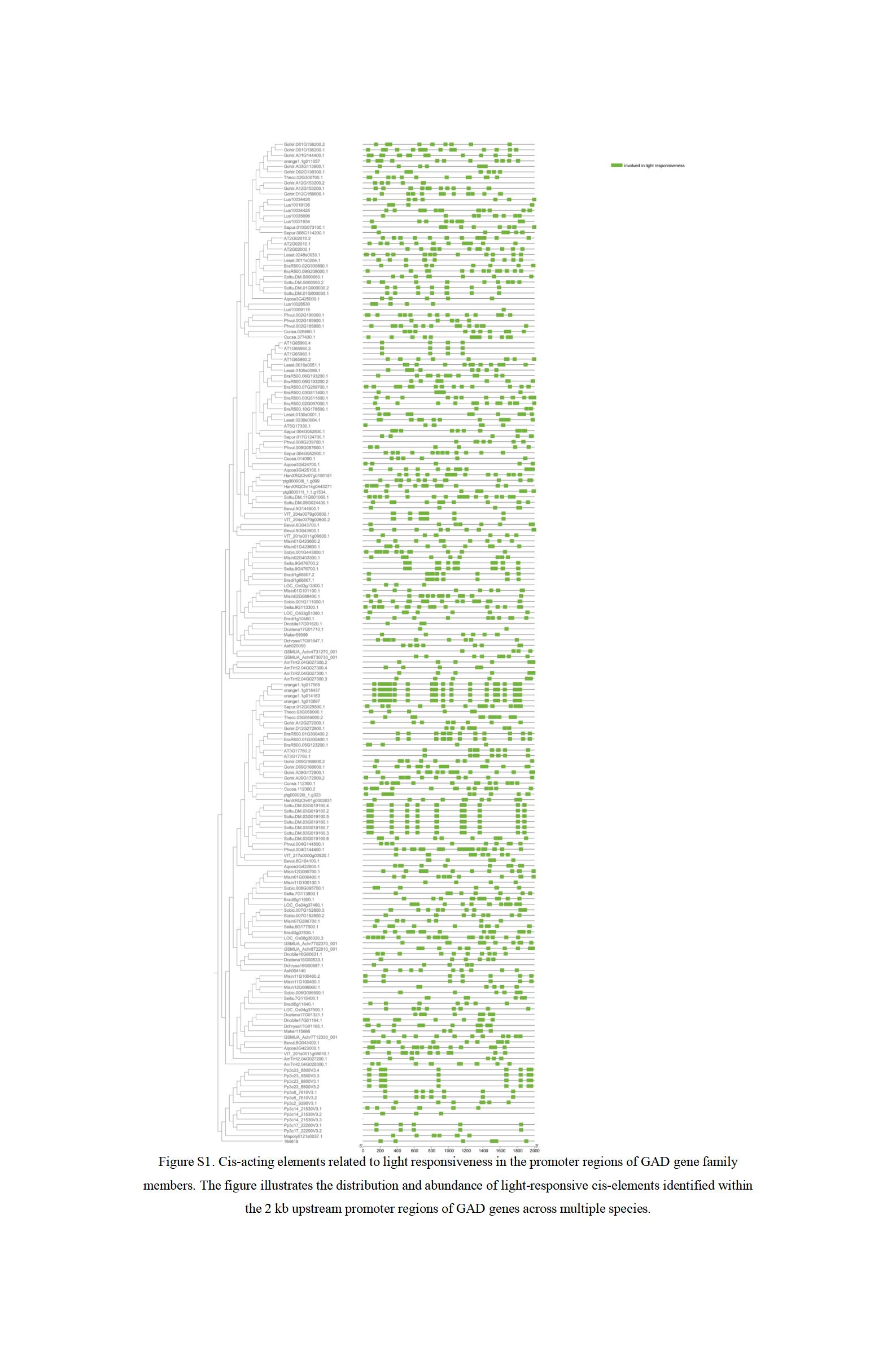

Supplement: Supplementary file 5 — Supplementary Material 5. [file 12870_2025_7691_MOESM5_ESM.jpg]

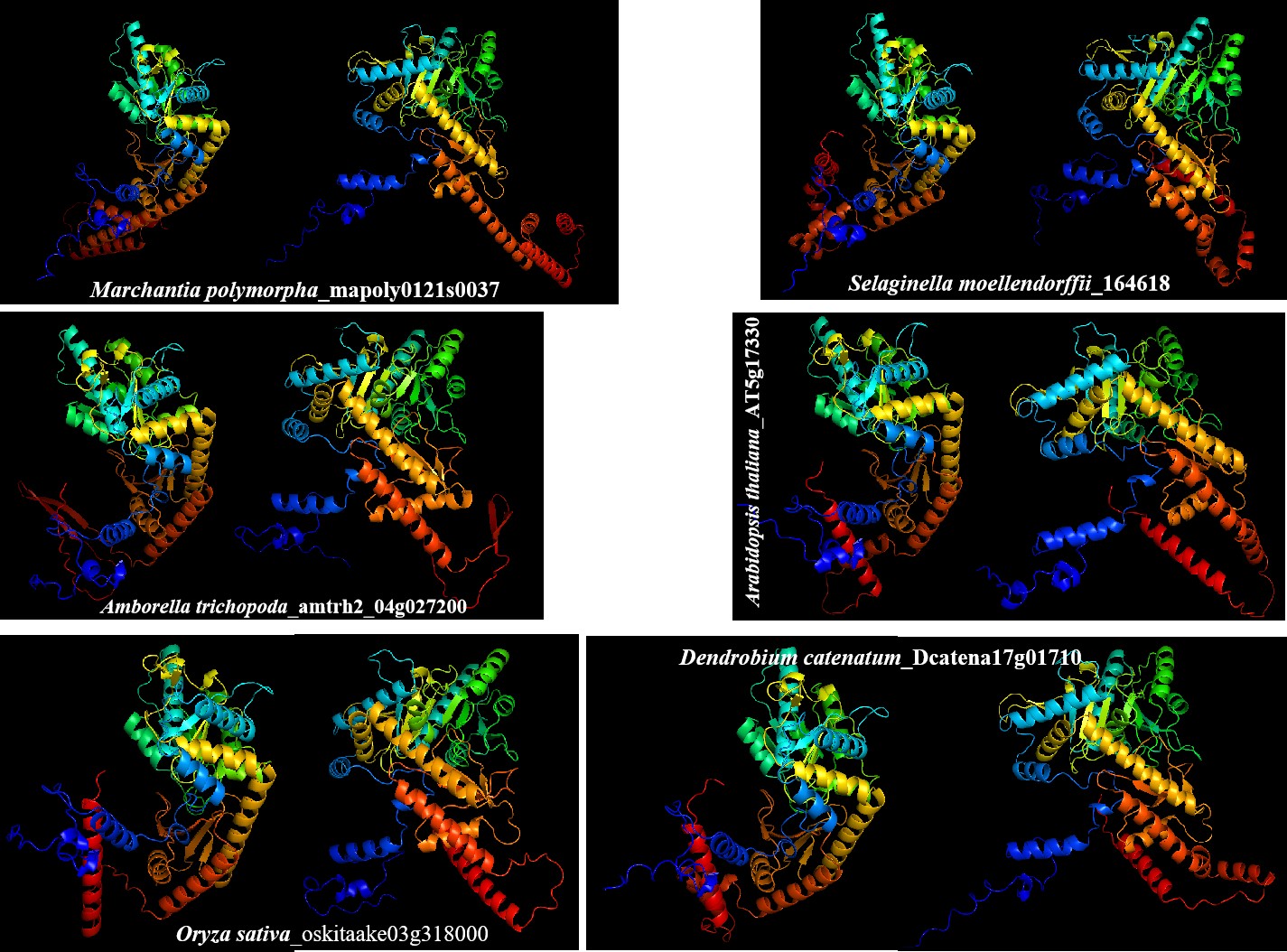

Supplement: Supplementary file 6 — Supplementary Material 6. [file 12870_2025_7691_MOESM6_ESM.jpg]
